# Domestic violence against women in Turkey

**DOI:** 10.12669/pjms.343.15139

**Published:** 2018

**Authors:** Fatma Basar, Nurdan Demirci

**Affiliations:** 1 Fatma Basar, PhD, Assistant Professor, Department of Obstetrics and Gynecology Nursing, Dumlupinar University, Kutahya School of Health, Kutahya, Turkey; 2 Prof. Nurdan Demirci, PhD. Marmara University, Faculty of Health Sciences, Nursing Department of Gynecology and Obstetrics, Istanbul, Turkey

**Keywords:** Affecting factors, Domestic violence, Prevalence

## Abstract

**Objective:**

To identify the prevalence of domestic violence and the factors that influence domestic violence.

**Methods:**

A cross-sectional descriptive research design was used with data from 1481 women over 18 years of age who were married at least once. Demographics and the Scale for Domestic Violence against Women (SDVAW) were used to collect the data.

**Results:**

The results showed that 41.3% of the women experienced domestic violence, and the majority (89.2%) had been subjected to violence by their spouses. There was a significant relationship between the mean SDVAW score and educational status, income status, spouse’s age, spouse’s education level, marriage age, family type and subjection to violence (p<.05).

**Conclusion:**

Low social status in Turkey was connected to the level of domestic violence. Efforts should be made to improve women’s social status. Changing the country’s patriarchal system, and educating women and their spouses can be useful in preventing domestic violence.

## INTRODUCTION

Domestic violence is an important public health problem worldwide, regardless of geographical limits, economic development, and educational level and affects women of all ages. Domestic violence is usually carried out by the spouse, with women typically being the recipients.^1^,^2^ Violence against women causes many problems, including physical injury, impaired mental health and quality of life, chronic pain, disability, suicide attempts, drug and alcohol use, depression, nightmares, and social isolation. Being a victim of domestic violence increases the rate of utilization of health services and affects the mental health of family members.^3^,^4^ While rates of violence against women in the 21st century are increasing, studies on the prevalence of and factors affecting it in Turkey are minimal. It is expected that the conclusions of the present study will contribute to enlarging the literature on domestic violence and promoting efforts to prevent violence against women. In addition, the findings of this study could confer specific goals to the Turkey National Plan for Violence against Women, which needs a stronger focus on the prevention of violence. For this reason, in the case of Turkey, it is necessary to understand the different dimensions of violence thoroughly and analyze their different aspects. This is the first study conducted throughout the city of the prevalence of domestic violence against women in Kutahya, the forms of violence and risk factors. The results of this study are important for leading the way, as a data source, for efforts to prevent violence against women and future studies. This study aimed to identify the prevalence of domestic violence and the factors that influence domestic violence.

## METHODS

The population of the study comprised women over 18 years old in the Central Kutahya District (Central Kutahya District N = 90.198 according to the Turkish Statistical Institute Address Based Population Registration System, 2013). The sample size was calculated as 383 using the number of individuals per house and the known sample spread formula (confidence interval: 95%, margin of error: 5%, sample size: 383). However, considering that a larger sample size can contribute to study reliability, the sample was taken with 1481 women. Women over 18 years of age who had been married at least once were included in the study. After the calculation of sample size, stratified sampling was used to generalize the study result to Kutahya as a whole. Based on the city where the study was conducted, the population of women older than 18 years registered at all family health centers was determined. There are 24 family health centers in Kutahya. Each Family Health Center was regarded as one of 24 strata. The women included in the sample were determined using randomization tables. Women selected for the study, which were registered at family health centers were reached in their homes and invited to participate in the study.

The data were collected from April 1, 2015 to April 28, 2016. Demographics and the SDVAW were used to collect the data. Demographics form consists of questions concerning the socio-demographic and marital characteristics of the participants. Also the form contains questions about the occurrence of and type of violence to which the participants were subjected. The SDVAW was developed by Kilic in 1998 to measure the level and dimensions of domestic violence against women by their spouses. The scale consist of 50 items and 5 subscales. Subscales are physical violence, emotional violence; verbal violence, economic violence and sexual violence. All of the subscales consist of ten items, each with a minimum score of 1 and a maximum score of 30. All items are rated on a three-point Likert-type scale (1 = Never, 2 = Sometimes, 3 = Always). However, 16 statements were reverse coded. The lowest possible score for the whole scale is 50, whereas the highest score is 150. The total score obtained from the whole scale indicates the level of domestic violence against women. High scores that women get from the scale show high level of exposure to violence while low scores indicate low level of exposure to violence.^5^

Alpha values for the SDVAW and subgroups ranged from 0.73 to 0.94. In this study, the SDVAW had a Cronbach’s alpha of 0.90. For the subscales, the Cronbach’s alpha values were found to be 0.79 for physical violence, 0.60 for emotional violence, 0.72 for verbal violence, 0.68 for economic violence, and 0.72 for sexual violence.

### Statistical Analysis

In data analysis, SPSS Statistic Version 22.0 (SPSS, Inc., Chicago, IL, USA) was used. Descriptive data are presented as number, percentage, and mean. The data collected from the groups were compared by t-test and one-way ANOVA; a *p* value of <0.05 and a *p* value of <0.001 were considered significant.

## RESULTS

Of the women, 32.7% were aged between 28 and 37. The majority of women were primary school graduates (49.5%) and were not employed (72%); 75.4% of the respondents had a moderate income. The spouses of the women were older (32.7% over 48 years old), 31.3% were primary school graduates, and 93.3% were employed. In addition, 61.1% of the women were married at ages 11–20 years and 85.8% of them were in nuclear-type families (Table-I).

**Table-I:** Sociodemographic characteristics of women (N=1481).

Variables		n	%
Age (39.42 ±12.13) Min: 18 Max: 78	Ages 18–27	255	17.2
	Ages 28–37	485	32.7
	Ages 38–47	369	24.9
	48 and above	372	25.1
Education status	Elementary School	733	49.5
	Secondary School	184	12.4
	High School	300	20.3
	College and higher education	264	17.8
Profession	Employed	414	28.0
	Unemployed	1067	72.0
Income status	Income less than expenditure	196	13.2
	Income equal to expenditure	1116	75.4
	Income more than expenditure	169	11.4
Age of the Spouse	Ages 18–27	128	8.9
	Ages 28–37	420	29.1
	Ages 38-47	422	29.3
	48 and above	472	32.7
Education status of the spouse	Elementary School	455	31.3
	Secondary School	219	15.1
	High School	437	30.1
	College and higher education	343	23.6
Marriage age	Ages 11–20	905	61.1
	Ages 21–23	284	19.2
	Ages 24–26	205	13.8
	27 and above	87	5.9
Family type	Core family	1266	85.5
	Large family	176	11.9
	Scattered family	39	2.6
Experienced domestic violence	Yes	611	41.3
	No	870	58.7

*Total*		1481	100.0

Six hundred and eleven 41.3% of the women were subjected to domestic violence, 89.2% of them were subjected to violence by their spouses, and 67.9% of them did not respond to violence. Of the women exposed to violence, 44.8% were exposed to physical violence, 67.7% to emotional violence, 13.4% to sexual violence, 74.3% to verbal violence, and 18.5% to economic violence (Table-II).

**Table-II:** Women’s experience of domestic violence.

		n	%
Domestic violence cases (n=1481)	Yes	611	41.3
	No	870	58.7
Types of violence (n = 611)			
Physical violence	Yes	274	44.8
	No	337	55.2
Emotional violence	Yes	417	67.7
	No	194	23.3
Sexual violence	Yes	82	13.4
	No	529	86.6
Verbal violence	Yes	457	74.3
	No	154	26.7
Economic violence	Yes	113	18.5
	No	498	81.5
Other violence	Yes	6	1.0
	No	605	99.0
The person carrying out the violence (n = 611)	Spouse	545	89.2
	Mother/Father	43	7.0
	Sibling	2	0.3
	Mother-in-law/Father-in-law	12	2.0
	Friend/colleague	9	1.5
The frequency of being subjected to violence (n = 611)	Always	158	25.9
	Sometimes	364	59.6
	Rarely	89	14.6
The reaction given when subjected to violence* (n = 611)	I did not respond	415	67.9
	I received an apology and made peace	208	34.0
	I went to the police station	68	11.1
	I left the house	75	12.3
	I responded	69	11.2

*Total*		1481	100.0

The total mean score for the SDVAW scale was 69.12 ± 12.10; for the subscales, the mean scores were found to be 10.95 ± 2.09 for the physical violence subscale, 16.22 ± 3.05 for emotional violence, 15.43 ± 3.25 for verbal violence, 14.04 ± 3.20 for economic violence, and 12.71 ± 2.78 for sexual violence (Table-III).

**Table-III:** Scale of domestic violence against women scores averages of women.

SDVAW Subscale	X̄ ± SD	Lower and Upper Values	Min.	Max.
Physical violence	10.95 ± 2.09	10–30	10	30
Emotional violence	16.22 ± 3.05	10–30	10	28
Verbal violence	15.43 ± 3.25	10–30	10	30
Economic Violence	14.04 ± 3.20	10–30	10	27
Sexual violence	12.71 ± 2.78	10–30	10	29
Total Score	69.12 ± 12.10	50–150	50	143

There was a statistically significant relationship between the mean scores of the women on the SDVAW and their educational level, income status, spouse’s age, spouse’s educational level, marriage age, family type and subjection to violence (p<0.05). There was no statistically significant relationship between the mean SDVAW scores and age, profession, and spouse’s profession (p>0.05) (Table-IV).

**Table-IV:** Factors affecting Scale of domestic violence against women scores.

Variables		X̄ ± SD	t/F	p
Age	Ages 18–27	67.95 ± 0.71		
	Ages 28–37	69.01 ± 0.69	0.926	0.428
	Ages 38–47	69.55 ± 0.78		
	48 and above	69.81 ± 0.86		
Education status	Elementary School	71.36 ± 13.73		
	Secondary School	71.11 ± 11.71	16.174	0.000
	High School	67.59 ± 10.14		
	College and higher education	65.01 ± 8.81		
Profession	Employed	68.61 ± 12.75	-0.970	0.332
	Unemployed	69.40 ± 11.74		
Income status	Income less than expenditure	75.76 ± 1.47		
	Income equal to expenditure	68.62 ± 0.42	21.079	0.000
	Income more than expenditure	66.38 ± 0.91		
Age of the spouse	Ages 18–27	67.13 ± 7.98		
	Ages 28–37	67.41 ± 9.64	3.570	0.014
	Ages 38–47	70.15 ± 13.21		
	48 and above	69.47 ± 13.00		
Education status of the spouse	Elementary School	71.21 ± 12.23		
	Secondary School	71.41 ± 12.83	16.006	0.000
	High School	69.03 ± 12.53		
	College and higher education	64.96 ± 8.72		
Spouse’s Profession	Employed	68.75 ± 11.61	-0.564	0.573
	Unemployed	69.30 ± 12.72		
Marriage age	Ages 11–20	70.66 ± 13.35		
	Ages 21–23	66.58 ± 8.72	7.749	0.000
	Ages 24–26	67.08 ± 11.26		
	27 and above	68.00 ± 8.89		
Family type	Core family	68.26 ± 10.88		
	Large family	72.36 ± 14.42	22.935	0.000
	Scattered family	81.75 ± 22.79		
Experienced	Yes	76.13 ± 14.65	14.665	0.000
Domestic violence	No	64.49 ± 6.89		

## DISCUSSION

In the present study, 41.3% of the women had been subjected to domestic violence (74.3% verbal, 67.7% emotional and 44.8% physical) and the mean scores of the SDVAW were found to be high. Studies conducted in Turkey have reported that from 13% to 78% of women are subjected to domestic violence at some point in their lives.^6^,^7^ Castro RJ et al.(2017) in Peru found that 38.5% of women had been subjected to violence.^8^ According to 2012 WHO data, 13-61% of the women are exposed to physical violence, 6-59% to sexual violence and 20-75% to emotional violence by their partners.^9^ Other studies in the literature show that women are subjected to high rates of domestic violence.^1^,^10^,^11^ often by their spouses.^1^,^12^ The results of the present study showed that the rate domestic violence against women in Turkey is as high as in other countries.

This study found that low educational level, low income, young marriage age, fragmented family type, spouse’s age, low educational level of spouse and subjection to violence were associated with domestic violence. Jahromi MK et al. (2016) found that low levels of education were associated with domestic violence in a study carried out to determine the factors related to domestic violence for women in Iran.^11^ Other studies have reported that domestic violence is associated with a low education level.^12^-^15^. As can be seen in the results of this study, a low level of education is associated with domestic violence. Thus, since the level of domestic violence has been found to decrease as the educational level increases, it is suggested that if the educational level of women is increased, domestic violence would be reduced.

The mean SDVAW score for women with low incomes was found to be higher than the score for those with higher incomes. Similarly, Barnawi1 (2015) reported that a low income level is associated with domestic violence.^1^ There are similar studies in the literature reporting that low income level affects domestic violence.^16^,^17^ Because low income levels are thought to lead to various family problems, it can be stated that the low socioeconomic level has an important relationship with the level of domestic violence. However, it can also be considered that women with a high income may speak less of the violence they experience because they think they could harm themselves or their careers.

There was relationship between spouse’s age and domestic violence. The mean SDVAW score in the older spouses group (38–47) was higher and are exposed to violence more than the others. Similar to the present study, Fageeh (2014) noted that the spouses of women who are exposed to violence are older than those of women who are not exposed to violence.^13^ Barnawi also found that advanced age and exposure to domestic violence were related.^1^ It could be stated that people can conceal and control violence at a younger age, but they cannot hide violence at an older age and reveal it. Studies in the literature suggest that there is an important relationship between early marriage age and domestic violence.^1^,^12^,^15^

The present study found a statistically significant relationship between marriage age and experience of domestic violence. The results of this study show that early marriage age is associated with higher incidence of domestic violence. Early marriages are common in Turkey. In countries such as Turkey, education and employment opportunities can be improved by ensuring that the marriage age of women is higher, which may decrease the level of domestic violence towards women.

Family type is also among the risk factors for domestic violence.^3^In this study, a statistically significant relationship was found between family type and the mean SDVAW score for respondents with a scattered family was higher. Vest JR et al. (2002) found that divorce or separate living were associated with domestic violence.^16^ In the scattered family type, family integrity is impaired and disagreements are more frequent, affecting the level of domestic violence.

In addition, the present study found that those who were exposed to violence obtained a higher mean score on the scale of attitudes toward violence. Studies on violence report that witnessing or experiencing violence can be influential on individuals’ attitudes towards violence. The reason for this may be that violence is a learned behavior, and is transferred to future generations in this way.

## CONCLUSION

The results of this study show that domestic violence is still a major problem in Turkey. Nearly half of the women had suffered domestic violence and most of them have been subjected to violence by their husbands. Low social status in Turkey was connected to the level of domestic violence. Efforts should be made to improve women’s social status. Changing the country’s patriarchal system, and educating women and their spouses can be useful in preventing domestic violence.

### Authors’ Contributions

**FB:** Conceived, designed, did data collection, did statistical analysis, writing of manuscript, takes responsibility for integrity of research work. **ND:** Planning the study.
